# Zoonotic Tuberculosis as a One Health Challenge: Global Evidence, Transmission Dynamics, and Policy Gaps in Indonesia

**DOI:** 10.3390/vetsci13030237

**Published:** 2026-02-28

**Authors:** Tyagita Hartady, Faisal Amri Satrio, Syahrul Maulana, Dwi Wahyuda Wira, Endang Yuni Setyowati, Annas Salleh

**Affiliations:** 1 Study Program of Veterinary Medicine, Faculty of Medicine, Padjadjaran University, Sumedang 45363, Indonesia; f.a.satrio@unpad.ac.id (F.A.S.); syahrul22001@mail.unpad.ac.id (S.M.); dwi.wahyudha@unpad.ac.id (D.W.W.); endang.yuni@unpad.ac.id (E.Y.S.); 2 Department of Biomedical Science, Faculty of Medicine, Padjadjaran University, Sumedang 45363, Indonesia; 3 Department of Animal Production, Faculty of Animal Husbandry, Padjadjaran University, Sumedang 45363, Indonesia; 4 Department of Veterinary Laboratory Diagnosis, Faculty of Veterinary Medicine, Universiti Putra Malaysia, Serdang 43400, Selangor, Malaysia; annas@upm.edu.my

**Keywords:** livestock diseases, molecular epidemiology, *Mycobacterium bovis*, One Health, public health, zoonotic tuberculosis

## Abstract

**Highlights:**

**What are the main findings?**

- Zoonotic tuberculosis (*Mycobacterium bovis*) is underdiagnosed and underreported in LMICs.
- zTB likely exceeds the estimated 1–1.5% of global human TB cases due to limited diagnostics.
- Rising bovine TB, raw milk consumption, and informal slaughtering increase human exposure in Indonesia.
- Livestock co-infections complicate zTB detection and may enhance bacterial shedding.
- Strengthened One Health surveillance, diagnostics, and food-safety systems are critical for zTB control.

**What are the implications of the main findings?**

The findings highlight zoonotic tuberculosis as a substantially underrecognized contributor to the tuberculosis burden in low- and middle-income countries, with Indonesia representing a high-risk setting due to increasing bovine TB prevalence, raw milk consumption, informal slaughtering practices, and weak food-safety oversight. Underdiagnosis driven by limited molecular diagnostic capacity and fragmented human–animal surveillance systems likely obscures the true extent of *Mycobacterium bovis* transmission to humans. Co-infections in livestock further compromise disease detection and may enhance pathogen shedding, increasing exposure risk along dairy and beef value chains. These challenges underscore the urgent need for strengthened One Health approaches that integrate public health, veterinary, and food-safety systems, supported by improved laboratory capacity, coordinated surveillance, and targeted risk mitigation measures to reduce zoonotic TB transmission and support national TB control goals.

**Simple Summary:**

Zoonotic tuberculosis (zTB), mainly caused by *Mycobacterium bovis*, is an underdiagnosed yet important disease transmitted from animals to humans through the consumption of raw milk, contaminated animal products, and close contact with infected livestock. Global estimates suggest that zTB accounts for approximately 1–1.5% of human tuberculosis cases; however, the true burden is likely underestimated due to limited diagnostic capacity, particularly in low- and middle-income countries. In Indonesia, increasing bovine tuberculosis cases in regions such as West Java, East Java, and Bali, combined with weak food-safety practices and limited access to molecular diagnostics, heighten the risk of undetected zoonotic transmission. Co-infections in livestock further complicate diagnosis and control efforts. This review summarises the global and Indonesian epidemiology of zTB, outlines transmission pathways at the human–animal interface, examines associations between zTB and other livestock infections, and identifies key gaps in surveillance and diagnostic capacity. Finally, it underscores the need for strengthened One Health approaches in Indonesia, including enhanced laboratory infrastructure, improved coordination between public and animal health sectors, and stronger food-safety regulation to mitigate zTB risk.

**Abstract:**

Zoonotic tuberculosis (zTB), predominantly caused by *Mycobacterium bovis*, remains an underrecognized public health threat in many low- and middle-income countries. Although global estimates suggest that zTB accounts for approximately 1–1.5% of all human tuberculosis cases, limited molecular diagnostic capacity and underreporting likely obscure its true burden. In Southeast Asia, particularly Indonesia, increasing detection of bovine tuberculosis in dairy and beef production systems—combined with high rates of raw milk consumption, informal slaughtering practices, and weak intersectoral surveillance—may amplify the risk of human exposure. Co-infections in livestock, including mastitis and respiratory pathogens, further complicate clinical detection and may enhance bacterial shedding. This review synthesises global and national epidemiological patterns of zTB, describes major transmission pathways at the human–animal interface, and examines interactions between *M. bovis* infection and other livestock diseases. Critical gaps in diagnostics, surveillance integration, and food-safety regulation are identified. Strengthening One Health approaches through improved laboratory capacity, coordinated public–animal health systems, and enhanced risk mitigation along dairy and beef value chains is essential to reduce the burden of zTB in Indonesia.

## 1. Introduction

Tuberculosis (TB) remains one of the most devastating infectious diseases affecting humanity, with profound impacts on public health, socioeconomic development, and global health security [1]. Despite decades of concerted international control efforts, TB continues to cause substantial morbidity and mortality, particularly in low- and middle-income countries (LMICs) [2,3]. According to the World Health Organization (WHO), an estimated 10.6 million people developed TB globally in 2023, with approximately 1.3 million deaths attributed to the disease, making TB the leading cause of death from a single infectious agent worldwide [4]. These figures underscore the persistent gap between global TB elimination targets and current epidemiological realities.

The overwhelming majority of human TB cases are caused by *Mycobacterium tuberculosis*, a pathogen highly adapted to human hosts and primarily transmitted via airborne droplets [5,6,7]. However, zoonotic tuberculosis (zTB), defined as TB caused by members of the *Mycobacterium tuberculosis* complex (MTBC) transmitted between animals and humans, represents a neglected yet clinically and epidemiologically significant component of the global TB burden [8,9,10]. zTB is most commonly associated with *Mycobacterium bovis (M. bovis)*, the principal causative agent of bovine tuberculosis. However, increasing evidence highlights the role of other MTBC members, including *M. caprae*, *M. africanum*, and *M. orygis* [11,12].

Historically, *M. bovis* was a major cause of human TB before the widespread introduction of milk pasteurization, meat inspection, and systematic bovine TB control programs in high-income countries [13,14]. These interventions led to dramatic declines in zTB incidence across Europe and North America during the twentieth century. Nevertheless, zTB remains endemic in many LMICs, where livestock production systems are characterized by close human–animal contact, limited veterinary oversight, informal slaughter practices, and inadequate food-safety regulations [15]. In such settings, livestock are integral not only to food security but also to cultural identity, social capital, and household income, amplifying the public health implications of animal diseases.

In animals, bovine TB causes chronic disease characterized by weight loss, reduced productivity, infertility, and increased mortality [16]. Economic consequences include decreased milk and meat production, trade restrictions, and financial losses associated with carcass condemnation [17]. These impacts disproportionately affect smallholder farmers, perpetuating cycles of poverty and vulnerability [18]. In humans, zTB contributes to both pulmonary and extrapulmonary diseases, often with diagnostic delays and suboptimal treatment outcomes [19].

Despite its recognized zoonotic potential, the true contribution of zTB to the global TB epidemic remains poorly quantified. WHO estimates suggest that zTB accounts for approximately 1–2% of all human TB cases worldwide, corresponding to an estimated 140,000 new cases and 12,000 deaths annually. However, these figures likely represent substantial underestimates due to diagnostic limitations, lack of routine MTBC speciation, fragmented surveillance systems, and weak coordination between the public health and veterinary sectors [20]. In many endemic countries, human TB diagnostics focus on detecting MTBC without species differentiation, whereas veterinary surveillance for bovine TB is sporadic or absent.

The challenge of zTB is further compounded by the global crisis of antimicrobial resistance (AMR). Drug-resistant TB threatens the effectiveness of current treatment regimens and undermines the progress toward TB elimination [21]. While AMR is most commonly discussed in the context of *M. tuberculosis*, the resistance dynamics in zTB are equally concerning [22]. *M. bovis* is intrinsically resistant to pyrazinamide, a cornerstone of first-line TB therapy, and can acquire additional resistance through chromosomal mutations under antimicrobial pressure [23]. The intersection of zTB and AMR highlights the need for integrated cross-sectoral responses.

Indonesia represents a particularly important yet understudied setting for zTB. The country consistently ranks among nations with the highest TB burden globally and hosts one of the largest livestock populations in Southeast Asia [24]. Livestock production in Indonesia is dominated by smallholder and backyard systems in which cattle, buffaloes, goats, and poultry are raised in proximity to human dwellings. Informal milk distribution, limited pasteurization, and uneven enforcement of slaughterhouse regulations create favorable conditions for zoonotic transmission [25,26]. Nevertheless, zTB remains largely absent from national TB control priorities and policy discourse.

The One Health approach, which recognizes the interconnectedness of human, animal, and environmental health, provides a comprehensive framework for addressing zTB [27]. By integrating surveillance, diagnostics, policy, and community engagement across sectors, the One Health strategy offers a pathway to more effective and sustainable control. This review synthesizes global evidence on zTB, examines available data and policy contexts from Indonesia, explores the emerging challenge of AMR, and identifies strategic opportunities for implementing One Health-based interventions at the human–livestock interface.

## 2. Materials and Methods

### 2.1. Study Design and Conceptual Framework

This study was designed as a One Health-oriented integrative original research study, combining systematic evidence synthesis, comparative epidemiological analysis, and policy evaluation to characterize zTB agents, transmission pathways, antimicrobial resistance (AMR) mechanisms, and surveillance gaps, with a focused institutional and regulatory assessment in Indonesia. The analytical framework integrated human, animal, and environmental health components in accordance with the established One Health principles [13,17]. This study aimed to generate actionable insights to strengthen zTB control strategies and antimicrobial resistance mitigation efforts.

### 2.2. Data Sources and Literature Identification

Primary data sources included peer-reviewed scientific literature indexed in PubMed, Scopus, and Web of Science, as well as surveillance reports and policy documents from the World Health Organization (WHO), Food and Agriculture Organization (FAO), World Organisation for Animal Health (WOAH; formerly OIE), and relevant Indonesian government agencies. Additional sources included molecular epidemiology and whole-genome sequencing (WGS) studies of the *Mycobacterium tuberculosis* complex (MTBC). Search terms were applied in various combinations and included “zoonotic tuberculosis,” “*Mycobacterium bovis*,” “*Mycobacterium orygis*,” “MTBC speciation,” “bovine tuberculosis,” “antimicrobial resistance,” “One Health,” and “Indonesia.” The literature search covered publications from 1990 to 2025, capturing both historical tuberculosis control programs and recent advances in molecular epidemiology.

### 2.3. Eligibility Criteria

The included studies met at least one of the following criteria: identification or characterization of zoonotic MTBC members; analysis of transmission pathways between humans, livestock, wildlife, or the environment; molecular or phenotypic characterization of AMR in MTBC; and evaluation of TB diagnostics, surveillance systems, or policy frameworks.

Exclusion criteria included studies unrelated to MTBC and non-systematic opinion pieces without empirical or policy relevance.

### 2.4. Data Extraction and Analytical Approach

Data were extracted into structured matrices capturing MTBC species and host range, transmission routes, drug resistance determinants, diagnostic approaches, and surveillance and policy characteristics. Qualitative synthesis was complemented by a comparative regional analysis (LMICs versus high-income countries) and a case study of Indonesia.

### 2.5. Manuscript Preparation

During the preparation of this manuscript, the authors used ChatGPT (GPT-5.2; OpenAI, San Fransisco, CA, USA) for the purposes of generating the draft text, data collection, and assisting in the conceptualization of graphical elements. The authors have reviewed and edited the output and take full responsibility for the content of this publication.

## 3. Results and Discussion

### 3.1. Etiology and Diversity of zTB Agents

Members of the *M. tuberculosis* complex cause zTB, a group of closely related mycobacteria that can infect humans and a wide range of domestic and wild animals. MTBC members share more than 99% genomic similarity, yet exhibit distinct host preferences, virulence profiles, and epidemiological characteristics. Understanding this diversity is essential for accurate diagnosis, surveillance, and control.

#### 3.1.1. *M. bovis* as the Principal Zoonotic Agent

*M. bovis* is the most important zTB agent and the primary cause of bovine tuberculosis worldwide. Unlike *Mycobacterium tuberculosis*, which is highly adapted to humans, *M. bovis* exhibits a broad host range, infecting cattle, buffaloes, goats, pigs, deer, a wide range of wildlife species, and humans [28]. This ecological versatility facilitates cross-species transmission and complicates eradication efforts.

From a clinical perspective, *M. bovis* infection in humans is indistinguishable from *M. tuberculosis* infection based on clinical presentation, radiological findings, and routine microbiological diagnostics [29]. However, a critical therapeutic distinction is that *M. bovis* is intrinsically resistant to pyrazinamide (PZA), a first-line anti-tuberculosis drug that is essential for shortening the treatment duration [30]. This resistance results from mutations or deletions in pncA, which encodes pyrazinamidase, the enzyme required to convert PZA into its active form, pyrazinoic acid [31]. Failure to correctly identify *M. bovis* infection may therefore lead to inappropriate treatment regimens, prolonged infectiousness, and an increased risk of treatment failure [32].

#### 3.1.2. Emerging Zoonotic MTBC Members

Beyond *M. bovis*, other MTBC members have increasingly been recognized as zoonotic pathogens. *M. caprae* has been reported in goats, cattle, and humans in several European countries and is often associated with small ruminant farming systems [33,34]. *M. africanum*, traditionally considered a human-adapted pathogen, has been detected in animal reservoirs and contributes substantially to the TB burden in West Africa [35,36].

Recently, *M. orygis* has emerged as a zoonotic agent of concern in South and Southeast Asia [37]. Initially identified in antelopes and cattle, *M. orygis* has since been isolated from patients with TB, particularly in India, Bangladesh, and neighboring regions [38,39]. Molecular epidemiological studies suggest bidirectional transmission between humans and livestock, challenging the traditional dichotomy between human and animal TB.

These findings highlight the limitations of focusing exclusively on *M. bovis* and underscore the need for comprehensive MTBC speciation in both human and animal TB surveillance systems.

### 3.2. Susceptibility to Secondary Bacterial Infections in Livestock Affected by zTB

zTB transmission occurs through complex and interrelated pathways, involving direct contact, environmental exposure, and foodborne routes. The relative importance of these pathways varies across settings, livestock management practices, and cultural behaviors.

#### 3.2.1. Foodborne Transmission of Zoonotic Tuberculosis via Unpasteurized Dairy Products

The ingestion of unpasteurized milk and dairy products from infected animals has historically been the most common route of zTB transmission to humans [40,41]. This route is frequently associated with extrapulmonary TB manifestations, including cervical lymphadenitis, gastrointestinal TB, genitourinary TB, and skeletal disease [42]. In many LMICs, the consumption of raw milk persists due to cultural preferences, perceived health benefits, lack of refrigeration, and limited access to pasteurized products.

#### 3.2.2. Aerosol Transmission and Occupational Exposure to Zoonotic Tuberculosis

Aerosol transmission plays a significant role in occupational settings. Farmers, abattoir workers, butchers, veterinarians, and livestock traders are at an increased risk of inhaling infectious aerosols during close contact with infected animals, carcasses, or contaminated environments [43]. Pulmonary TB caused by *M. bovis* is clinically indistinguishable from *M. tuberculosis*, often leading to misclassification and underreporting [44]. *M. bovis* pulmonary infection is a form of pulmonary tuberculosis that is indistinguishable from other forms of the disease clinically, radiographically, and symptomatically, including chronic cough, fever, and weight loss hemopstysis, as well as characteristic changes in chest imaging. Since the standard diagnostic algorithm used in most tuberculosis control programmes is based on smear microscopy, “undifferentiating” GeneXpert assays, or culture without species-level identification, *M. bovis* infections are frequently reported in error as *M. tuberculosis* cases. This diagnostic shortcoming, combined with a lack of access to mycobacterial speciation and molecular typing in many low- and middle-income areas, contributes significantly to the under-recognition and underreporting of zoonotic tuberculosis.

#### 3.2.3. Transmission of Tuberculosis in Livestock and Wildlife Reservoirs

**Figure 1:**
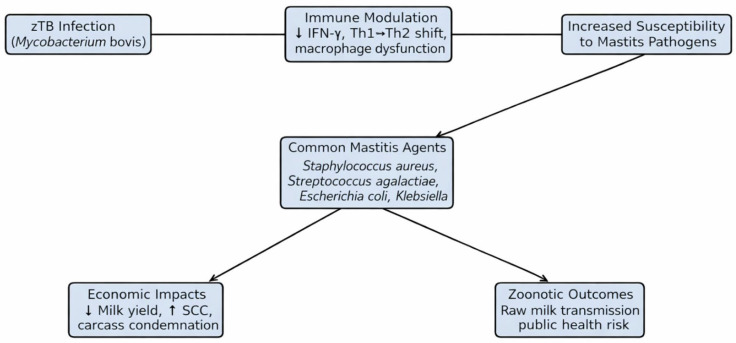
Proposed mechanistic pathway linking zoonotic tuberculosis (*M. bovis*) infection in livestock to increased susceptibility to secondary bacterial mastitis and its downstream impacts. Initial infection with *M. bovis* can impair host immunity by reducing interferon-gamma (IFN-γ) production, shifting the immune profile from a Th1-dominated to Th2-biased response, and reducing macrophage bactericidal capacity. This immune modulation predisposes the mammary gland to colonization by common mastitis pathogens, including *Staphylococcus aureus*, *Streptococcus agalactiae*, *Escherichia coli*, and *Klebsiella pneumoniae*. The resulting co-infection can lead to elevated somatic cell counts, reduced milk yield, carcass condemnation, and prolonged treatment durations, while also creating a zoonotic risk via raw milk consumption. Integrating bTB surveillance with mastitis control strategies is essential in endemic areas to break this cycle of immune compromise and economic loss. Notes: ↓ Milk yield means milk production decreases. ↑ SCC → The upward arrow means somatic cell count (SCC) increases.

In livestock, TB transmission occurs primarily via the respiratory route, facilitated by close confinement, poor ventilation, and high stocking densities [45]. Wildlife reservoirs, such as badgers in the United Kingdom, white-tailed deer in North America, and wild boars in Europe, play a critical role in maintaining infection cycles and reintroducing TB into livestock populations [46]. These multihost systems pose major challenges for eradication (Table 1 and Figure 1).

#### 3.2.4. Environmental Persistence

MTBC organisms can persist in soil, water, and organic matter for extended periods under favorable conditions, particularly in tropical environments characterized by moderate temperatures and high humidity [47]. Environmental persistence may enable indirect transmission and complicate control efforts, especially in extensive farming systems [48,49].

### 3.3. Global Epidemiology and Burden of zTB

Estimating the global burden of zTB remains a challenge. WHO estimates suggest that zTB accounts for approximately 1–2% of all human TB cases globally, corresponding to roughly 140,000 new cases and 12,000 deaths annually. However, these figures likely represent substantial underestimates.

#### 3.3.1. Regional Patterns

Sub-Saharan Africa bears a disproportionate burden of zTB, driven by endemic bovine TB, extensive livestock dependence, and high HIV prevalence [50,51]. In South Asia, the increasing detection of *M. orygis* and *M. bovis* highlights the diversity of zTB agents and transmission pathways [37,39].

In high-income countries, the incidence of zTB has declined owing to long-standing bovine TB control programs and food-safety regulations. Nevertheless, sporadic cases persist, often linked to wildlife reservoirs, migration, or consumption of unregulated animal products [50,52] (Table 2 and Table 3).

#### 3.3.2. Vulnerable Populations

ZTB disproportionately affects marginalized populations, including rural farmers, informal slaughterhouse workers, and communities with limited access to healthcare [54]. Socioeconomic factors, such as poverty, malnutrition, and HIV co-infection, exacerbate susceptibility and disease severity [55].

### 3.4. Antimicrobial Resistance and zTB

#### 3.4.1. Molecular Basis of Antimicrobial Resistance in MTBC

Antimicrobial resistance (AMR) in the M. tuberculosis complex arises primarily through chromosomal mutations rather than horizontal gene transfer, distinguishing mycobacteria from many other bacterial pathogens [56]. These mutations alter drug targets, metabolic pathways, and drug activation processes, thereby reducing drug susceptibility [57,58].

*M. bovis* exhibits intrinsic resistance to pyrazinamide (PZA), a key first-line anti-TB drug [59,60,61]. This resistance is attributed to mutations or deletions in the pncA gene, which encodes pyrazinamidase—an enzyme required for the conversion of the prodrug pyrazinamide to its active form, pyrazinoic acid [62]. As a result, standard six-month TB regimens are ineffective against *M. bovis*, necessitating longer and modified treatment protocols.

Beyond intrinsic resistance, *M. bovis* and other zoonotic MTBC members can acquire resistance through point mutations in genes encoding drug targets (Table 4):

- Isoniazid resistance is commonly associated with mutations in the katG gene (notably Ser315Thr), which impair prodrug activation, and in the inhA promoter region, leading to target overexpression [63]
- Rifampicin resistance arises from mutations in the rpoB gene, particularly within the rifampicin resistance-determining region (RRDR), resulting in drug binding to RNA polymerase [64]
- Streptomycin resistance is linked to mutations in rpsL and rrs, affecting ribosomal protein S12 and 16S rRNA, respectively [65].

Whole-genome sequencing (WGS) studies increasingly demonstrate that resistance-conferring mutations in *M. bovis* mirror those observed in *M. tuberculosis*, confirming shared evolutionary pathways under antimicrobial pressure [66] (Table 4).

#### 3.4.2. Zoonotic Transmission of Drug-Resistant Strains

Although historically considered rare, drug-resistant zTB is increasingly being documented. Reports from Europe, Africa, and Latin America have described *M. bovis* isolates resistant to isoniazid, rifampicin, and ethambutol in both humans and cattle [67,68]. Molecular epidemiological studies have confirmed the bidirectional transmission of resistant strains between livestock and humans, particularly in settings with close occupational contact [69,70].

The public health implications of this study are significant. Misclassification of zTB as drug-susceptible *M. tuberculosis* may lead to treatment failure, prolonged infectiousness, and an increased risk of resistance amplification. In addition, the undetected transmission of resistant strains within livestock populations can act as a silent reservoir, undermining TB elimination efforts.

#### 3.4.3. Livestock Antimicrobial Use and Selection Pressure

Although first-line anti-TB drugs are not authorized for use in livestock, indirect antimicrobial pressure plays a crucial role in the emergence of resistance. In many LMICs, broad-spectrum antibiotics such as tetracyclines, fluoroquinolones, and aminoglycosides are extensively used in animal husbandry for disease prevention and growth promotion [71,72].

Environmental contamination of soil, water, and animal microbiota with antimicrobial residues creates selective pressure that may facilitate the survival and persistence of resistant mycobacterial populations [73,74]. Fluoroquinolone resistance is of particular concern, as these drugs are critical components of multidrug-resistant TB (MDR-TB) treatment regimens in humans [75,76]. Experimental studies have demonstrated that subinhibitory antimicrobial exposure can induce stress responses and mutagenesis in mycobacteria, potentially accelerating the development of resistance [77].

#### 3.4.4. Implications for One Health AMR Control

The convergence of zTB and antimicrobial resistance (AMR) underscores the urgency of integrating antimicrobial stewardship into the One Health framework. Coordinated surveillance of drug resistance across human and animal health sectors, combined with molecular typing and whole-genome sequencing (WGS), is essential for identifying transmission pathways and emerging resistance hotspots [78,79]. Failure to address AMR in the context of zTB risks undermines global tuberculosis elimination targets and further exacerbates the already substantial burden of drug-resistant TB.

### 3.5. Diagnostic Gaps and Fragmented One Health Surveillance in zTB Control

Routine tuberculosis diagnostics are capable of detecting members of the *Mycobacterium tuberculosis* complex (MTBC) but do not differentiate species [80]. Species-level identification requires culture-based methods and advanced molecular techniques, which remain inaccessible in many low-income and middle-income countries [81]. Veterinary surveillance for bovine tuberculosis is often limited, and human and animal health systems frequently operate in silos, constraining integrated detection and response efforts [82].

### 3.6. zTB in Indonesia: Policy, Regulatory, and Institutional Context

#### 3.6.1. National TB Control Framework

Indonesia’s TB control efforts are primarily guided by the National TB Control Program (NTP) under the Ministry of Health, which is aligned with the WHO End TB Strategy. The NTP focuses predominantly on *M. tuberculosis* transmission in humans, with a strong emphasis on case detection, standardized treatment, and management of drug-resistant TB.

However, zTB is not explicitly addressed in the national TB guidelines. Diagnostic algorithms rely heavily on Gene Xpert MTB/RIF, which detects MTBC but does not differentiate species, resulting in systematic under-recognition of *M. bovis* and other zoonotic MTBC infections [83].

#### 3.6.2. Veterinary and Livestock Health Regulations

Animal health governance in Indonesia is overseen by the Ministry of Agriculture, and bTB is recognized as a notifiable disease under veterinary law. However, the implementation of surveillance and control measures remains inconsistent. Economic constraints, lack of compensation mechanisms, and resistance from smallholder farmers constrain test-and-slaughter programs [84].

Routine ante-mortem and post-mortem inspections in abattoirs vary in quality, particularly in informal slaughter settings where most rural livestock processing occurs [85]. Molecular confirmation of bTB is rarely performed, and data sharing between the veterinary and public health sectors is minimal [86].

#### 3.6.3. Antimicrobial Use and Stewardship Policy in Indonesia

Indonesia has taken steps to address antimicrobial resistance through the National Action Plan on Antimicrobial Resistance (NAP-AMR), which formally adopts the One Health Approach [87]. The plan includes objectives related to antimicrobial stewardship, surveillance, and public awareness across both the human and animal health sectors [88,89].

Despite this policy framework, its enforcement remains weak. Over-the-counter access to antibiotics for livestock is common, and veterinary oversight is limited, particularly in smallholder and backyard farming systems [90,91]. Surveillance of antimicrobial resistance in animal pathogens rarely includes mycobacteria, creating a critical blind spot in efforts to address zoonotic tuberculosis-associated resistance.

#### 3.6.4. One Health Institutional Challenges

Although Indonesia has formally endorsed the One Health principles, operationalization remains fragmented. Human health, veterinary services, and environmental agencies operate largely in silos, with limited data integration and joint risk assessment [92,93].

zTB exemplifies these challenges. The absence of joint surveillance systems, limited laboratory capacity for MTBC speciation, and a lack of cross-sectoral training hinder effective control. Strengthening interministerial coordination, investing in diagnostic infrastructure, and integrating zTB into NTP and veterinary disease control programs are critical priorities.

#### 3.6.5. Strategic Opportunities for Indonesia

Indonesia is well-positioned to strengthen zTB control through the following measures:

- Integration of MTBC speciation into reference laboratories, with targeted surveillance in high-risk occupational groups;
- Expansion of AMR monitoring to include zoonotic pathogens, with community education on food safety and occupational risk;
- Strengthening compensation mechanisms to support livestock disease reporting.

Such measures would significantly advance Indonesia’s commitment to the One Health initiative and contribute to global efforts to eliminate TB.

### 3.7. One Health Challenges and Strategic Opportunities

zTB exemplifies the critical importance of the One Health approach, which integrates human, animal, and environmental health, particularly at the human–animal interface, where the risk of transmission is greatest [94,95,96]. The epidemiology of zTB is shaped by the combined influence of livestock production systems, food handling and processing practices, occupational exposure, and the environmental persistence of members of the *Mycobacterium tuberculosis* complex (MTBC). In many low- and middle-income settings, including Indonesia, smallholder farming systems, close human–animal contact, inadequate milk pasteurization, and limited biosecurity measures collectively facilitate ongoing zoonotic transmission and the perpetuation of zTB.

A key priority within the One Health framework is strengthening harmonized laboratory capacity. The limited availability of diagnostic tools capable of discriminating *M. bovis* and other zoonotic members of the *Mycobacterium tuberculosis* complex (MTBC) from *Mycobacterium tuberculosis* has contributed to substantial underreporting due to the misdiagnosis of zTB cases [97,98]. Expanding access to molecular diagnostic tools, including PCR-based assays and whole-genome sequencing, would substantially improve the early detection and accurate identification of zTB.

Integrating zTB into national tuberculosis control programmes represents a critical opportunity to strengthen TB prevention and control. Currently, most TB programs remain largely focused on human-to-human transmission, with limited consideration of animal reservoirs or zoonotic sources of infection [99]. The incorporation of *zTB* into TB control frameworks would enable the systematic collection of animal contact information during case and contact investigations, targeted screening of high-risk occupational groups—including farmers, abattoir and meat-processing workers, and veterinarians—and the adaptation of treatment guidelines to account for *zTB*-specific considerations, particularly in settings where *M. bovis* circulation is documented.

Strengthening veterinary surveillance is equally critical to the successful implementation of a One Health approach. In many endemic countries, surveillance for bovine tuberculosis remains limited and is often largely reliant on abattoir-based monitoring [100]. Enhancing veterinary surveillance systems would enable earlier detection of infection in cattle and reduce the risk of spillover transmission to humans. However, the absence of adequate compensation schemes for livestock culled as part of disease control programmes represents a major barrier to compliance among cattle owners. Strengthening veterinary services, together with the introduction of effective compensation mechanisms, would therefore support improved management of zoonotic transmission of *M. bovis* and enhance bovine tuberculosis control in endemic settings.

Antimicrobial stewardship represents a cross-cutting priority within the One Health framework, as inappropriate antimicrobial use in both the human and animal health sectors can indirectly undermine tuberculosis control efforts. Although *M. bovis* is intrinsically resistant to pyrazinamide, misdiagnosis of zTB and the use of standard treatment regimens may result in suboptimal clinical outcomes and prolonged infection [101]. Effective antimicrobial stewardship, together with appropriate diagnostic and treatment practices, is essential to ensure optimal case management while minimizing unnecessary selective pressure on members of the *Mycobacterium tuberculosis* complex.

Finally, community engagement and risk communication are essential for sustainable zTB control. Education programs promoting milk pasteurization, safe meat handling, improved animal husbandry, and occupational protection have been shown to be effective in lowering zoonotic transmission rates [102]. In Indonesia, strategic opportunities exist through networks in the local community, farmer cooperatives, and religious and traditional leaders to convert the principles of One Health into culturally sensitive and context-specific interventions. Altogether, these strategic opportunities point out that the effective control of zTB is not just a question of technical and biomedical solutions but requires sustained political will, intersectoral coordination, and community involvement based on a strong One Health approach.

## 4. Discussion

zTB is a persistent yet under-recognized component of the global tuberculosis burden, arising from diverse members of the *Mycobacterium tuberculosis* complex that circulate across human, livestock, wildlife, and environmental reservoirs. This study highlights that zTB is sustained through interconnected foodborne, aerosol, occupational, and wildlife-mediated transmission pathways, disproportionately affecting marginalized populations in low- and middle-income countries. The intrinsic resistance of *M. bovis* to pyrazinamide, together with the increasing detection of acquired resistance among zoonotic MTBC members, underscores the critical association between zTB and antimicrobial resistance.

Diagnostic limitations, particularly the lack of routine MTBC speciation, continue to drive the underestimation of zTB and inappropriate treatment. The case study from Indonesia illustrates how fragmented surveillance systems, limited veterinary control measures, and weak intersectoral coordination hinder effective detection and response, despite formal commitments to the One Health approach and antimicrobial resistance mitigation. Addressing zTB requires integrated One Health strategies that align human and animal health surveillance, strengthen laboratory capacity for species-level diagnosis, and embed antimicrobial stewardship across sectors. Recognising zTB as a core component of national tuberculosis control programmes is essential to achieving sustainable tuberculosis elimination and mitigating the growing threat of drug-resistant disease.

## Figures and Tables

**Table 1:** Risk factors facilitating respiratory transmission of tuberculosis in livestock systems.

Risk Factor	Mechanism of Transmission Enhancement	Production Systems Affected	Reference
High stocking density	Increased aerosol exposure	Intensive dairy and beef	[45]
Poor ventilation	Accumulation of infectious droplets	Confined housing systems	[45]
Prolonged animal contact	Sustained exposure duration	Feedlots and barns	[45]
Stress and immunosuppression	Increased susceptibility	Transport and fattening systems	[45]

**Table 2:** Regional patterns and drivers of zTB.

Region	Estimated zTB Burden	Dominant MTBC Species	Key Drivers	References
Sub-Saharan Africa	High	*M. bovis*	Endemic bovine TB, livestock dependence, and HIV prevalence	[50,51]
South Asia	Moderate (likely underestimated)	*M. orygis*, *M. bovis*	Close human–livestock contact and limited diagnostics	[38]
High-income countries	Low	*M. bovis*	Wildlife reservoirs, migration, and food practices	[11,53]

**Table 3:** Wildlife and food-associated sources of zTB in high-income countries.

Source	Transmission Route	Examples	Epidemiological Significance	Reference
Wildlife reservoirs	Direct and environmental contact	Badgers, deer, wild boar	Maintenance of infection cycles	[53]
Unregulated animal products	Oral ingestion	Raw milk, artisanal cheese	Sporadic human cases	[11]
Human migration	Reactivation or importation	Migrant populations	Surveillance challenge	[53]

**Table 4:** Genetic determinants of antimicrobial resistance in zoonotic MTBC members.

Anti-TB Drug	Primary Gene(s)	Resistance Mechanism	Zoonotic MTBC Relevance	Reference
Pyrazinamide	*pncA*	Loss of prodrug activation	Intrinsic resistance in *M. bovis*	[63]
Isoniazid	*katG*, *inhA* promoter	Impaired activation; target overexpression	Acquired resistance	[64]
Rifampicin	*rpoB* (RRDR)	Reduced RNA polymerase binding	Acquired resistance	[65]
Streptomycin	*rpsL*, *rrs*	Altered ribosomal binding	Acquired resistance	[66]

## Data Availability

The original contributions presented in this study are included in the article. Further inquiries can be directed to the corresponding author(s).

## Abbreviations

The following abbreviations are used in this manuscript:

bTB	Bovine tuberculosis
CTLA-4	Cytotoxic T-Lymphocyte-Associated Protein 4
IFN-γ	Interferon-gamma
IL-1β	Interleukin-1 beta
LAG-3	Lymphocyte Activation Gene 3
*M. bovis*	*Mycobacterium bovis*
MTBC	*Mycobacterium tuberculosis* complex
*M. tuberculosis*	*Mycobacterium tuberculosis*
OIE	World Organisation for Animal Health
PD-1	Programmed Cell Death Protein 1
PD-L1	Programmed Death-Ligand 1
SICCT	Single Intradermal Comparative Cervical Tuberculin Test
TB	Tuberculosis
Th-1	T helper-1
Th-2	T helper-2
TNF-α	Tumor Necrosis Factor-alpha
WHO	World Health Organization
zTB	Zoonotic tuberculosis
